# Human alveolar epithelial cells type II are capable of TGFβ-dependent epithelial-mesenchymal-transition and collagen-synthesis

**DOI:** 10.1186/s12931-018-0841-9

**Published:** 2018-07-24

**Authors:** Torsten Goldmann, Gernot Zissel, Henrik Watz, Daniel Drömann, Martin Reck, Christian Kugler, Klaus F. Rabe, Sebastian Marwitz

**Affiliations:** 1 Pathology of the University Medical Center Schleswig-Holstein (UKSH), Campus Lübeck and the Research Center Borstel, Parkallee 3a, 23845 Borstel, Germany; 2 Pneumology, University Medical Center, University of Freiburg, Hugstetter Straße 55, 79106 Freiburg, Germany; 3 Pulmonary Research Institute, Wöhrendamm 80, 22927 Großhansdorf, Germany; 4 Medical Clinic III: University Medical Center Schleswig-Holstein (UKSH), Campus Lübeck, Ratzeburger Allee 160, 23538 Lübeck, Germany; 5 Oncology, LungenClinic Grosshansdorf, Wöhrendamm 80, 22927 Großhansdorf, Germany; 6 Surgery, LungenClinic Grosshansdorf, Wöhrendamm 80, 22927 Großhansdorf, Germany; 7 Großhansdorf Pneumology, LungenClinic Grosshansdorf, Wöhrendamm 80, 22927 Großhansdorf, Germany; 8 Airway Research Center North Member of the German Center for Lung Research (DZL), Großhansdorf, Germany

**Keywords:** EMT, Alveolar epithelial cells, Fibrosis, Collagen

## Abstract

**Background:**

The origin of collagen-producing cells in lung fibrosis is unclear. The involvement of embryonic signaling pathways has been acknowledged and trans-differentiation of epithelial cells is discussed critically. The work presented here investigates the role of TGFB in cytoskeleton remodeling and the expression of Epithelial-Mesenchymal-Transition markers by Alveolar Epithelial Cells Type II and tests the hypothesis if human alveolar epithelial cells are capable of trans-differentiation and production of pro-fibrotic collagen.

**Methods:**

Primary human alveolar epithelial cells type II were extracted from donor tissues and stimulated with TGFβ and a TGFβ-inhibitor. Transcriptome and pathway analyses as well as validation of results on protein level were conducted.

**Results:**

A TGFβ-responsive fingerprint was found and investigated for mutual interactions. Interaction modules exhibited enrichment of genes that favor actin cytoskeleton remodeling, differentiation processes and collagen metabolism. Cross-validation of the TGFβ-responsive fingerprint in an independent IPF dataset revealed overlap of genes and supported the direction of regulated genes and TGFβ-specificity.

**Conclusions:**

Primary human alveolar epithelial cells type II seem undergo a TGFβ-dependent phenotypic change, exhibit differential expression of EMT markers in vitro and acquire the potential to produce collagen.

**Electronic supplementary material:**

The online version of this article (10.1186/s12931-018-0841-9) contains supplementary material, which is available to authorized users.

## Background

Idiopathic pulmonary fibrosis (IPF), a progressive disease of irreversible collagen-deposition in the alveolar interstitium, is regarded as the most aggressive form of diffuse parenchymal lung diseases (DPLD) with a median 3-year survival comparable to lung cancer [1]. IPF is mainly characterized by focal accumulation of fibroblasts, designated as fibroblast foci, hyperplasia and hyperthrophy of alveolar epithelial cells type II (AECII) as well as widespread fibrotic areas in the end-stage, designated as honeycombing, which massively impact normal lung physiology. Furthermore, acute exacerbations cause a rapid decline in lung function [2, 3] and anti-inflammatory or immunosuppressive therapies conceded little success [4]. The pathogenesis of IPF has been extensively reviewed [5, 6] and the transforming growth factor beta (TGFβ) pathway, among others, identified as a central driver of pathogenesis [7]. The TGFβ ligand has been localized at sites of extracellular matrix gene expression in IPF tissues [8] and deletion of the TGFβ receptor type II is protective against bleomycin-induced IPF in mice [9]. Myofibroblasts have long been attributed as the source of extracellular matrix (ECM) deposition during fibrogenesis but evidence from animal models and in vitro studies suggested an implication of AECII via epithelial mesenchymal transition [7, 10, 11], a developmental process that transiently shifts the cellular differentiation of an epithelial to a mesenchymal cell [12]. Despite studies using either rat or mouse AECII [13, 14] or immortalized cell lines [15] as well as isolated AECII from IPF patients [16], the direct mechanistic connection of the TGFβ pathway on trans-diffe rentiation and collagen metabolism in human, primary AECII remains to be elucidated. Hence, we therefore hypothesize that TGFβ stimulation of primary human AECII (hAECII) will induce downstream processes that might result in phenotypic changes which lead to differential expression of EMT markers as well as possible production of collagens. T.

## Methods

### Isolation of human alveolar epithelial cells type II (hAECII)

Tumor-free lung tissues from surgical specimens of 28 different lung cancer patients who underwent pneumectomy or lobectomy with curative intent at the LungenClinic Grosshansdorf were used for extraction of primary cells. The use of patient lungs for research purposes was approved by the ethics committee at the University of Lübeck (statement no. 07–157 and 14–043) and informed consent was retrieved. All experiments were performed in accordance with relevant guidelines and regulations.

The mean age of the donors (19 male and 9 female patients) was 66.9 years (+/− 9.4) at time of surgery. Please see Additional file 1: Table S4 for detailed characteristics. hAECII from fresh lung tissues were prepared as described elsewhere [17]. In short, lung tissue was cut into small pieces with a scalpel, washed with extraction buffer and incubated with Dispase II (Roche Applied Sciences, Mannheim, Germany) for 60 min. at 37 °C under constant stirring. Residual connective tissue and debris was removed by filtering through nylon gaze (100 μm, 50 μm and 20 μm). Resulting heterogeneous mixture of cells was centrifuged and the cell pellet resuspended in buffer with Accutase and DNAse and finally layered onto density gradient medium. Debris and erythrocytes were excluded by density gradient centrifugation and resulting cells placed onto petri dishes to remove fast-adhering cells (immune cells). Non-adhering cells were incubated with anti-CD45-paramagnetic microbeads (Miltenyi, Bergisch-Gladbach, Germany) to deplete CD45-positive immune cells via LD columns according to manufacturer’s instructions (Miltenyi, Bergisch-Gladbach, Germany) and retrieve untouched hAECII. Purity of extracted hAECII was assessed by two independent observers (SM, TG) on randomly selected, Hope-fixed and paraffin-embedded preparations where the hAECII-lineage marker TTF1, the surfactant molecule pro-SPC as well as the bronchial epithelial marker Podoplanin werestained by immunocytochemistry as described below. For means of quantification, the whole sample was visually analyzed by microscopy by two trained investigators (TG, SM) and the percentage of positive cells was assessed in 5% increments. The median TTF1-positivity from 11 different extractions was 90% as well as 95% for pro-SPC (*N* = 9), and 2.5% for Podoplanin (N = 9). See also Additional File 2: Figure S1. The resulting cells were counted and seeded in 24 well plates (for RNA isolation) or on cover-slips (for microscopy) at a density of 1 × 10^6^/Well and left over night for adherence prior to stimulation experiments.

### Stimulation with TGFβ1 and SB431542

Cell culture medium [RPMI1640 (PAN Biotech, Aidenbach, Germany) with 10% FCS and 1% penicillin/streptomycin (PAN Biotech, Aidenbach, Germany)] was changed to serum-free medium 4 h prior stimulation experiments. The cells were stimulated with 5 ng/ml of human recombinant TGFβ1 (Peprotech, Hamburg, Germany) as well as 10 μM of TGFβ receptor I kinase inhibitor SB431542 (Sigma Aldrich, Germany) for 48 h in an incubator with 37 °C and 5% CO_2_; each compound was reconstituted according to manufacturer’s instructions.

### Preparation of cell blocks

Cell blocks were prepared from unstimulated hAECII directly after extraction for assessment of extraction purity or from stimulated cells. After stimulation, hAECII were detached with Trypsin/EDTA, Hope-fixed, dehydrated and submerged in low-melting paraffin to produce cell blocks as described elsewhere [18].

### Immunocytochemistry

Immunocytochemistry from cell blocks was conducted as described elsewhere (Marwitz et al [18]. 2011) using mouse anti-E-Cadherin (1/400, Clone ECH6, Zytomed Systems, Germany), mouse anti TTF1 (1/100, Clone SPT24, Zytomed Systems, Germany), mouse anti-Podoplanin (1/100, Clone D2–40, Agilent Dako, Santa Clara, USA), rabbit anti-pro-SPC (1/100, polyclonal, ab90716, abcam, Cambridge, UK), mouse anti-Vimentin (1/1000, Clone V6, Zytomed Systems, Berlin, Germany) and polyclonal rabbit anti-Collagen I (1/1000, Abcam, Oxford, UK) diluted in antibody diluent (Zytomed Systems, Berlin, Germany). Negative controls were included under omission of primary antibody in every staining series.

### Visualization of actin cytoskeleton by Rhodamine-Phalloidin staining

Human AECII were seeded on cover-slips, stimulated for 48 h and subsequently fixed for 10 min. With 4% PFA. The PFA was removed and the cells were permeabilized with 0.1% Triton X-100 in PBS for 7 min at room-temperature (RT). After washing with PBS, a blocking step with 1% BSA/PBS was conducted for 20 min. to prevent unspecific binding followed by thorough washing with PBS. The actin-cytoskeleton was visualized by staining with 165 nM TRITC-conjugated phalloidin (Invitrogen, Carlsbad, CA, USA) and 1 μg/ml DAPI (Invitrogen, Carlsbad, CA, USA) diluted in antibody diluent (Zytomed Systems, Berlin, Germany) for 20 min. at RT. The solution was discarded and the cells washed with PBS prior to mounting on cover slides with DABCO (2.5% 1.4-diazabicyclo[2.2.2]octane in 90% glycerol). Images were taken at 200× magnification on a Nikon Eclipse 80i fluorescence microscope equipped with a CCD-camera. The actin signal per cell was quantified using ImageJ software with RGB modus. The specific actin signal was determined as the area of signal in the red channel and divided by number of nuclei (actin area/cell) in the image. All images were acquired at the same exposure time.

### Immunofluorescence staining

Fixation of cells was conducted as described above. Permeabilization was conducted with 0.25% Triton X-100/PBS for 7 min at RT. Double staining of target antigens was achieved by incubation with first primary antibody for 45 min. Followed by subsequent washing with PBS and incubation with the second primary antibody at the same conditions. Visualization of primary antibodies was conducted with Alexa488 or TRITC labeled secondary antibodies (Molecular Probes, Eugene, Oregon, USA) diluted at 1/200 with 1 μg/ml DAPI in antibody diluent (Zytomed Systems, Berlin, Germany) for 45 min. at room temperature. Unbound antibodies and DAPI were removed by washing with PBS before mounting of cover slips on microscopic slides (Super Frost Plus, Menzel Gläser, Germany) with DABCO. In general, each reagent step was followed by thorough washing with 200 μl of PBS. To detect cells of hAECII lineage, the surfactant protein transcription factor TTF-1 [19] was targeted with mouse anti-TTF-1 (clone SPT24, Zytomed Systems, Berlin, Germany) at 1/400 dilution. Activity of collagen production and secretion processes were assessed by targeting the collagen chaperon HSP47 [20] with rabbit anti-HSP47 (clone EPR4217, Abcam, Oxford, UK) at 1/100 dilution.

### In-cell western assay (ICW)

hAECII were seeded at a density of 1 × 10^5^/Well into 96 well plates and fixed after 48 h of stimulation with PFA at a final concentration of 2% (*v*/v) for 10 min at RT. The supernatant was removed and cells permeabilized with 50 μl of 0.25% Triton X-100/PBS for 7 min at RT. Supernatant was removed and cells were washed with 150 μl PBS/well three times. Unspecific binding of primary antibodies was reduced by 1.5 h of blocking with 150 μl 1× Rotiblock solution (Carl Roth, Karlsruhe, Germany) at RT. Blocking solution was discarded and cells incubated with 50 μl of primary antibodies diluted in 1× Rotiblock over-night at 4 °C. Background controls were included on each plate under omission of primary antibody to account for unspecific binding of secondary antibodies. The supernatant was removed next day and cells washed three times with 150 μl of PBS. Primary antibodies were detected with either goat anti rabbit IgG or goat anti mouse IgG conjugated with IRDye 800CW (Licor Biosciences, Bad Homburg, Germany) diluted 1/1200 in 50 μl of 1× Rotiblock and supplemented with 1/5000 diluted To-Pro3-Iodide (Thermo Fisher Scientific, Waltham, USA) for 1 h at RT in the dark. After incubation, the cells were washed three times with 150 μl PBS and remaining fluids discarded. 96 well plates were imaged on an Odyssey Clx near-infrared scanner (Licor Biosciences, Bad Homburg, Germany) at a resolution of 169 μm, auto scan intensity, low quality and a focus off-set of 3 mm. Data was analyzed with the Image Studio software version 4 (Licor Biosciences, Bad Homburg, Germany) and the ICW plug-in. A 96 well grid was placed on the image and intensity values for each well at 700 nm (To-Pro3; total cell signal) and 800 nm (IRDye 800CW) measured. Raw data was further processed to substract background signal (intensity values of IRDye 800CW without primary antibody) from each well of the 800 nm signal. The background-corrected 800 nm was then normalized to the 700 nm signal of each well to account for different cell numbers. Each experimental setting was carried out in triplicates. Mean values of each parameter were calculated and normalized to the expression value of the medium control.

### Sirius red/fast green collagen detection

For colorimetric quantification of collagen, the Sirius Red/Fast Green Collagen Staining Kit (Chondrex, Redmond, WA, USA) was used according to manufacturer’s instructions. Each assay was done on 1 × 10^6^ cells/well on a 24-well plate.

### Microarray analyses

Total RNA from 4 independent experiments of stimulated hAECII was extracted using the RNeasy Mini Kit (Qiagen, Hilden, Germany) according to manufacturer’s instructions. Integrity of extracted RNA was assessed on a Bioanalyzer (Agilent, Böblingen, Germany) with the Agilent RNA 6000 Nano kit. Transcription profiling of hAECII was conducted as described elsewhere [17]. The investigated data set has been deposited at Gene Expression Omnibus and is publicly accessible via GSE100854 upon publication. The datasat GSE10667 was used for validation of selected genes in human IPF and lung tissue samples.

### Statistical microarray analysis

For statistical analysis, hierarchical clustering and venn diagram analysis, GeneSpring software (Agilent, Böblingen, Germany) version 13 was used. Repeated Measures ANOVA with a Benjamini-Hochberg multiple testing correction cut-off of *p* ≤ 0.05 was used to compute significantly regulated genes (Additional file 3: Table S1). The venn diagram function of GeneSpring was used to compute overlaps of gene lists with a Fold Change of ≥2 compared to medium. From this, a list of differentially regulated entities (454) that were found to be exclusively up-regulated by TGFβ with a Fold Change of ≥2 was used as input for further experiments and named “TGFB fingerprint”. The MiSgDB V5.2 database online-tool of the BROAD Institute (http://software.broadinstitute.org/gsea/msigdb/index.jsp) was used to investigate the enrichment of significant Hallmark Gene Sets and Reactome pathways with a FDR q-value cut-off of *p* ≤ 0.05. The validation dataset from human IPF and lung tissues samples was imported into with the same pre-processing settings as the AECII data set GeneSpring and compromised probes were removed. Furthermore, a Moderated T Test with Benjamini-Hochberg Multiple Testing Correction (p ≤ 0.05) was computed to calculate differentially expressed genes between IPF and lung tissues. Those genes with a Log Fold Change ≥2 compared to lung tissues were selected for comparison with the TGFB fingerprint. Overlap of both dataset was analyzed by Venn diagram function and a list of consensus genes was generated. Here the Log Fold Changes of IPF compared to lung tissues as well as the stimulations of AECII compared to medium control were investigates with each other. In case of more than one probe per gene present in the list, the mean Log Fold Change was calculated and used.

### Network analysis of microarray data

For visualization and analysis of interaction networks and GO analysis, Cytoscape (V.3.4.) (http://www.cytoscape.org/) with the ReactomeFI plugin was used. For this, a list comprising 429 of the “TGFB fingerprint” probes with an annotated GeneSymbol was used to query the StringDB database for possible protein-protein interactions. Here, a confidence filter was applied which disregarded interactions with an interaction score of less than 0.4. Furthermore, non-linked entities were removed and only interacting proteins allowed in further analyses. The interaction score from the StringDB database was annotated to the “TGFB fingerprint” genes and imported into Cytoscape to display the database-derived interaction network. Here, the spectral partition based network clustering algorithm [21] from within the Reactome FIViz app was applied to derive modules of interacting proteins (See Additional File 4: Table S2). To obtain insight into possible underlying biological processes, each module was investigated for significant enrichment of GO terms from the Biological Process with a FDR cut-off of *p* ≤ 0.05 (Additional file 5: Table S3). The Top15 enriched GO terms per module according to their FDR *p*-value were retrieved and submitted to a word cloud generator (www.wortwolken.com). Here, the GO terms were analyzed for the frequency of used words with higher abundance encoded as enlarged size of the respective word.

### Data presentation and statistics

All bar charts or scatter dot plots from non-microarray experiments were drawn with GraphPad Prism Vers 7 (GraphPad, La Jolla, CA, USA). Data is shown as the mean value of biological replicates with error bars depicting the standard deviation. Repeated-Measures ANOVA for paired observations was used to test for statistical significance and Tukey’s Multiple Comparison Test. The number of biological replicates is shown in the figure legends. In all applied statistical tests *p* ≤ 0.05 (*), 0.01 (**) and 0.001 (***) were regarded as significant and figures labelled accordingly.

## Results

### TGFβ induces a distinct molecular transcription circuitry in alveolar epithelial cells type II that favors differentiation processes, actin cytoskeleton remodeling and collagen synthesis

To decipher the transcriptional response of hAECII to TGFβ, microarray experiments were conducted. hAECII isolated from 4 different patients were stimulated with 5 ng/ml TGFβ, 10 μM of the specific TGFβ-Receptor I Kinase Inhibitor SB431542, a combination of both or left unstimulated for means of medium control. SB431542 was included to assess the specificity of observed effects as exclusively caused by TGFβ. Therefore, any effect that would be induced or reduced by TGFβ stimulation is expected to be abolished upon pathway inhibition and hence, be regarded as TGFβ-specific.

**Fig. 1:**
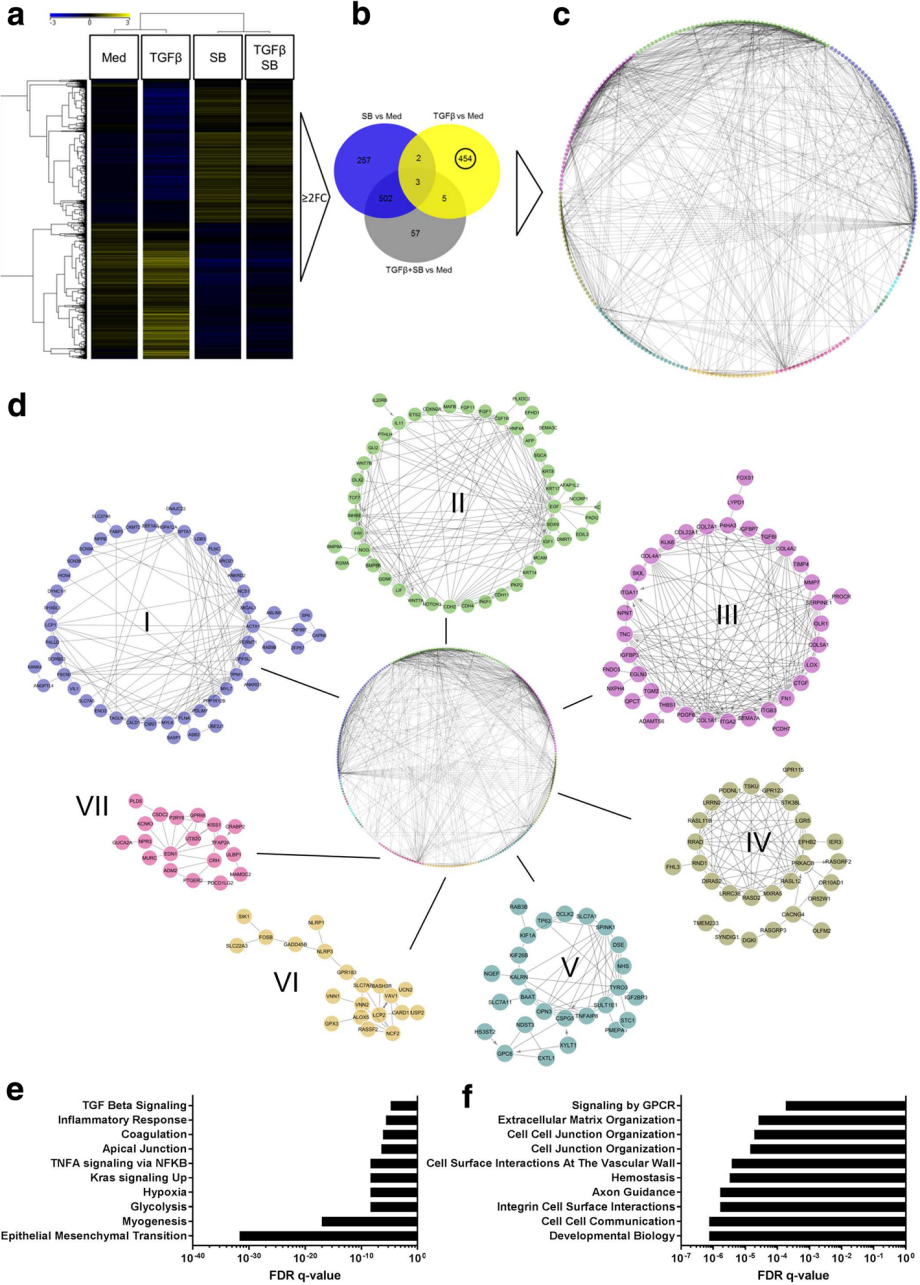
Transcriptional analysis of hAECII upon TGFβ stimulation. hAECII different donors were stimulated each with 5 ng/ml TGβ1, 10 μM SB431542, 5 ng/ml TGFβ1 and 10 μM SB431542 or left untreated for 48 h (*N* = 4). Significantly regulated genes were computed by Repeated-Measures ANOVA (RM-ANOVA) with a Benjamini-Hochberg multiple testing correction cut-off of ≤0.05. Averaged gene expression data of results from RM-ANOVA is shown as a Heatmap with Hierarchical Clustering of samples and entities according to Pearson Centered algorithm with Ward’s linkage rule. A Fold-Change Filter was applied to further analyze only probes that were at least ≥2 fold up-regulated compared to medium control (**a**). Out of those genes, a list of targets was extracted that was exclusively regulated by TGFβ and designated *TGFβ fingerprint* (**b**). The *TGFβ fingerprint* genes were further investigated for mutual interactions by querying the String protein-protein interaction database with those genes that exhibited an annotated GeneSymbol. A global map of these genes was constructed based on interaction scores within Cytoscape (**c**). The presence of intrinsic modules within the interactions of the *TGFβ fingerprint w*as computed by a spectral partition based cluster algorithm from the Reactome FIViz app and respective modules encoded by different colours. Further details of interactions within each module are shown in (**d**). Global enrichment of GSEA Hallmark gene sets (**e**) and Reactome pathways (**f**) from whole list of *TGFβ fingerprint* genes are displayed by their FDR q-value

After 48 h of incubation, RNA was extracted and subsequently used for whole genome gene expression analysis. A total of 10,284 genes were found to be significantly differential expressed among all experimental parameters (See Fig. 1a and Additional file 3: Table S1). These were further narrowed down to 1795 by filtering for a Fold Change (FC) ≥2 compared to medium control. To extract the exclusive effect of TGFβ stimulation on AECII, the lists of significantly regulated genes with a FC ≥ 2 were analyzed by using a Venn diagram comparing the experimental groups (Fig. 1b). 454 genes were found to be exclusively up-regulated by TGFβ in hAECII and not regulated by any other stimulation and hence, designated *TGFβ fingerprint*. 428 of these 454 hits exhibited an annotated GeneSymbol and were subjected to characterize the functional processes induced by TGFβ by means of network analysis. The String protein-protein interaction database was queried with the 428 GeneSymbols to derive potential protein-protein interaction networks within the *TGFβ fingerprint* of hAECII. The interaction network of the *TGFβ fingerprint* is depicted in a circular layout where spheres indicate individual genes/proteins and connecting lines mutual interactions (Fig. 1c). A spectral, partition-based network clustering algorithm [21] was applied to discover highly interacting modules within the network and highlighted by different sphere colours (See Additional file 4: Table S2 for genes attributed to each module). The 7 largest, by amount of involved members, were further selected and shown in Fig. 1d. Here, interacting genes and the proteins they account for could be assumed to share a similar biological effect or exert a similar biological function and process. First, the overall pathway enrichment of the *TGFβ fingerprint* was investigated for Hallmark Gene Sets and Reactome Pathways. The Top10 enriched pathway from each databases are depicted in Fig. 1e+F and display beside expected pathways as “TGFβ pathway” already very distinctive processes such as “Apical Junction”, “Myogenesis” or “Epithelial Mesenchymal Transition” (Fig. 1e) as well as several Hits from the Reactome database concerning cell-cell junctions or “Extracellular Matrix Organization” (Fig. 1f).

**Fig. 2:**
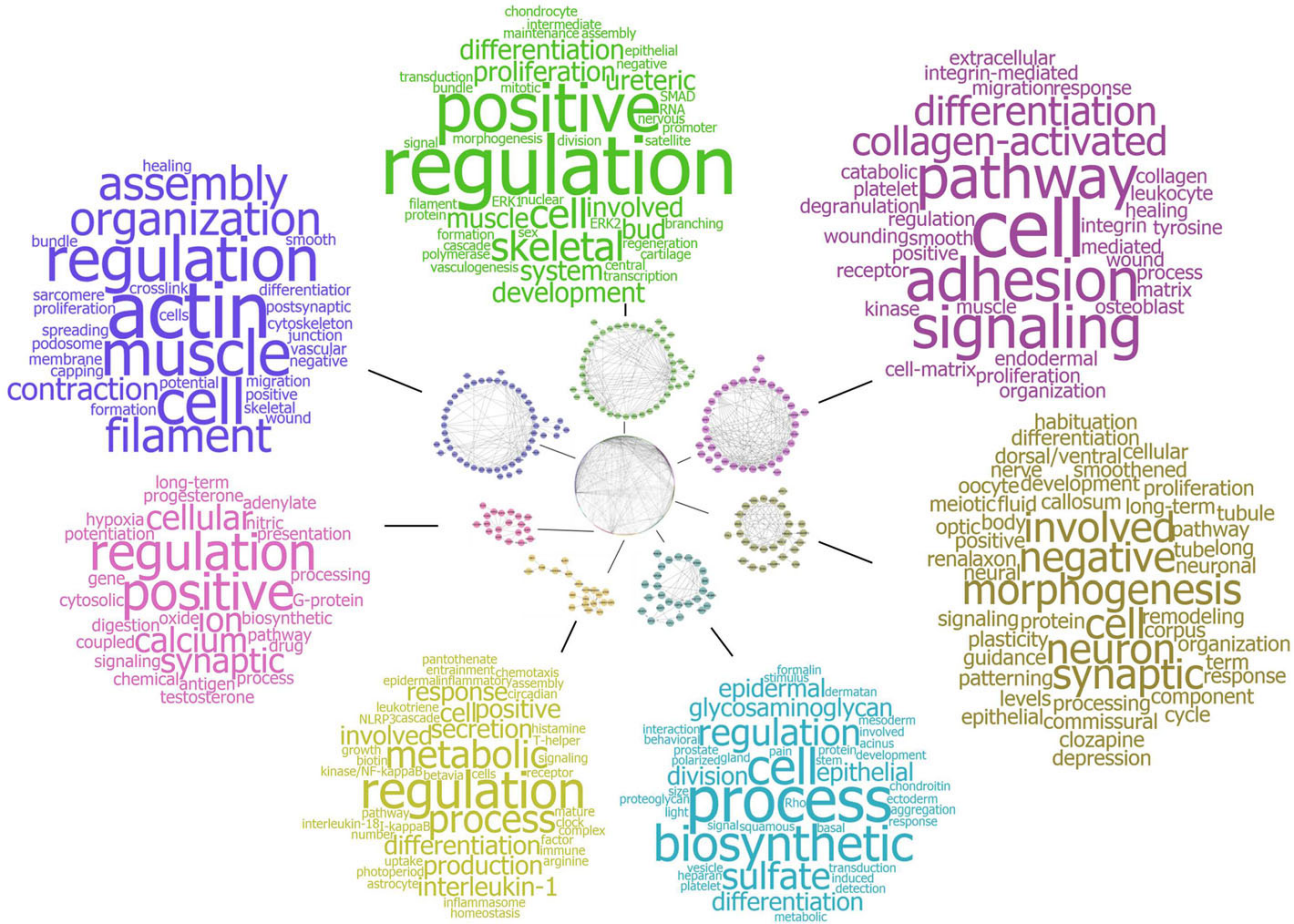
Word clouds of significantly enriched GO terms highlight activity of different biological processes governed by each module. Each module of the *TGFβ fingerprint* was investigated for significant enrichment of GO terms with a FDR cut-off of ≤0.05. The Top15 leading edge GO terms of Biological Process, by ranking of their corrected FDR *p*-value, were subjected to a word cloud generator for summary. Here, the relative abundance of each word within the submitted list of terms is directly related to the size within the word cloud. Words with a higher abundance exhibit an increased font size

To investigate the detailed biological role of each of the transcriptional modules within the *TGFβ fingerprint* interaction network, an enrichment of GO terms was calculated for each module and hence, the genes it accounts for. The Top15 leading edge GO terms, as indicated by ranking of the FDR *p*-values of enriched terms, were subjected to a word cloud generator and further summarized as a word cloud. Here, the abundance of each word from a list is directly connected to the size in the cloud with frequent words being depicted in larger font size (Fig. 2, See Additional file 5: Table S3 for total overview of significantly enriched GO terms). By this, each module of mutually interacting genes and their possible biological process is summarized by the words depicted in each cloud. Module I, which exhibits *ACTA1* as a central member with multiple interacting partners (Fig. 1d) is summarized by the words “actin”, “muscle”, “cell”, “filament” or “contraction” as the most prominent and frequent within the enriched GO terms. Module II which displays interconnected genes like *CDH2*, *FGF* or *EGF,* showed words and hence, GO terms, like “regulation”, “differentiation”, “development” or “cell” and hereby resembling module VII that exhibited comparable prominent words. Module III as the third largest, is constituted of genes coding for several collagens such as *COL1A1, COL4A2, COL4A1* or other extracellular matrix proteins such as fibronectin *FN1* and thereby leading to dominant words as “cell”, “adhesion”, “collagen-activated”, “wounding” or “differentiation”. The next 3 smaller modules, IV-VI exhibited dominant words as “morphogenesis”, “biosynthetic”, “process”, “glycosaminoglycan”, “metabolic”, “regulation” and “differentiation”. Since these observations were drawn from a limited number of patients, we further validated observed key processes for EMT in samples from independent patients on protein level.

### Human alveolar epithelial cells type II show TGFβ-dependent actin cytoskeleton remodeling

**Fig. 3:**
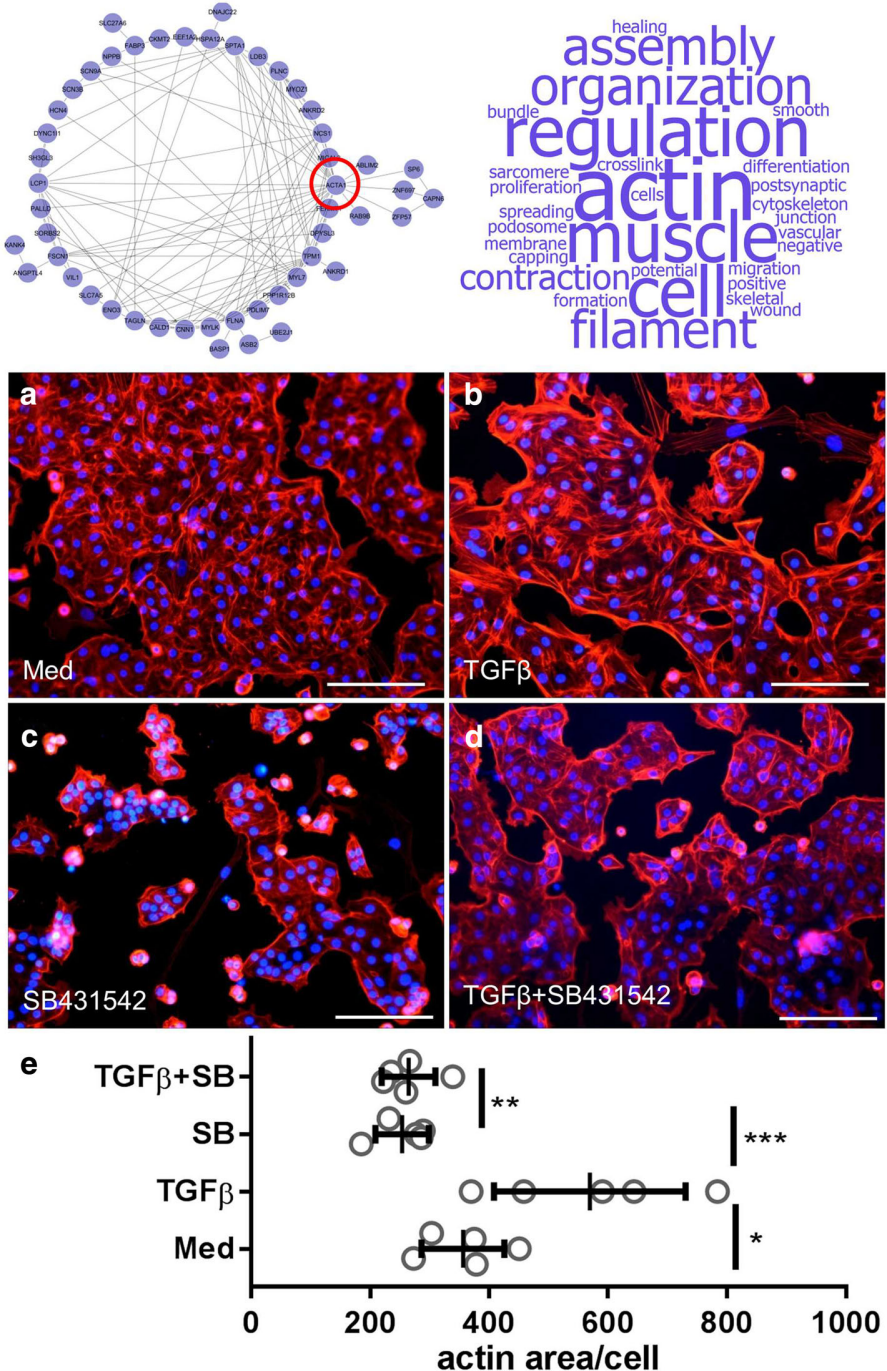
TGFβ induces actin cytoskeleton remodeling in hAECII. hAECII (*N* = 5) were seeded on coverslips and cultivated for 48 h in medium **a**, 5 ng/ml TGFβ **b**, 10 μM SB431542 (**c**) or both (**d**). The actin cytoskeleton was visualized by Rhodamine-Phalloidin and nuclei by DAPI. A representative image from one experiment shows the actin cytoskeleton at a magnification of 200× with a scale bar of 100 μm. Quantification of the actin area per cell was done with ImageJ and depicted as scatter dot plot (E) with the mean value and SD of 5 biological replicates. For statistical analysis, *p* ≤ 0.05 (*), 0.01 (**) and 0.001 (***) were regarded as significant

The network analysis and enrichment of GO terms of module I pointed towards processes which are constituted by elevated actin and filament organization. Therefore, remodeling of the actin-cytoskeleton network, which is regarded as a hallmark of cells during EMT [12, 15, 22], was assessed on protein level to validate the results from the gene expression arrays in cells from independent patients. During this process, a change in cell morphology and polarization is observed that is frequently accompanied by enhanced motility. To assess the overall change in cell morphology of hAECII upon stimulation with TGFβ, the actin filaments were visualized with Rhodamine-Phalloidin. TGFβ-specificity of observed effects was ensured by addition of TGFβ receptor I kinase inhibitor SB431542 [23]. 48 h post stimulation, which is a known timeframe to be sufficient for morphological changes during EMT, as induced by 5 ng/ml of TGFβ in A549 cells [15], strong actin signal was already observed in the medium control cells (Fig. 3a). Upon addition of 5 ng/ml, an increase in actin staining was observed with a distinct, filamentous morphology (Fig. 3b). Inhibition of the TGFβRI kinase itself resulted in dramatic reduction of the baseline actin signal (Fig. 3c) and stimulation with both, TGFβ and SB431542 did not result in an increased actin signal (Fig. 3d). Statistical evaluation of the measured actin area per cell resulted in a significant increase upon TGFβ stimulation, which was found to be successfully abolished upon addition of SB431542 as indicated by significant decreased actin area per cell (Fig. 3e).

### Human alveolar epithelial cells type II express EMT markers and display an elevated collagen metabolism in a TGFβ-dependent manner

**Fig. 4:**
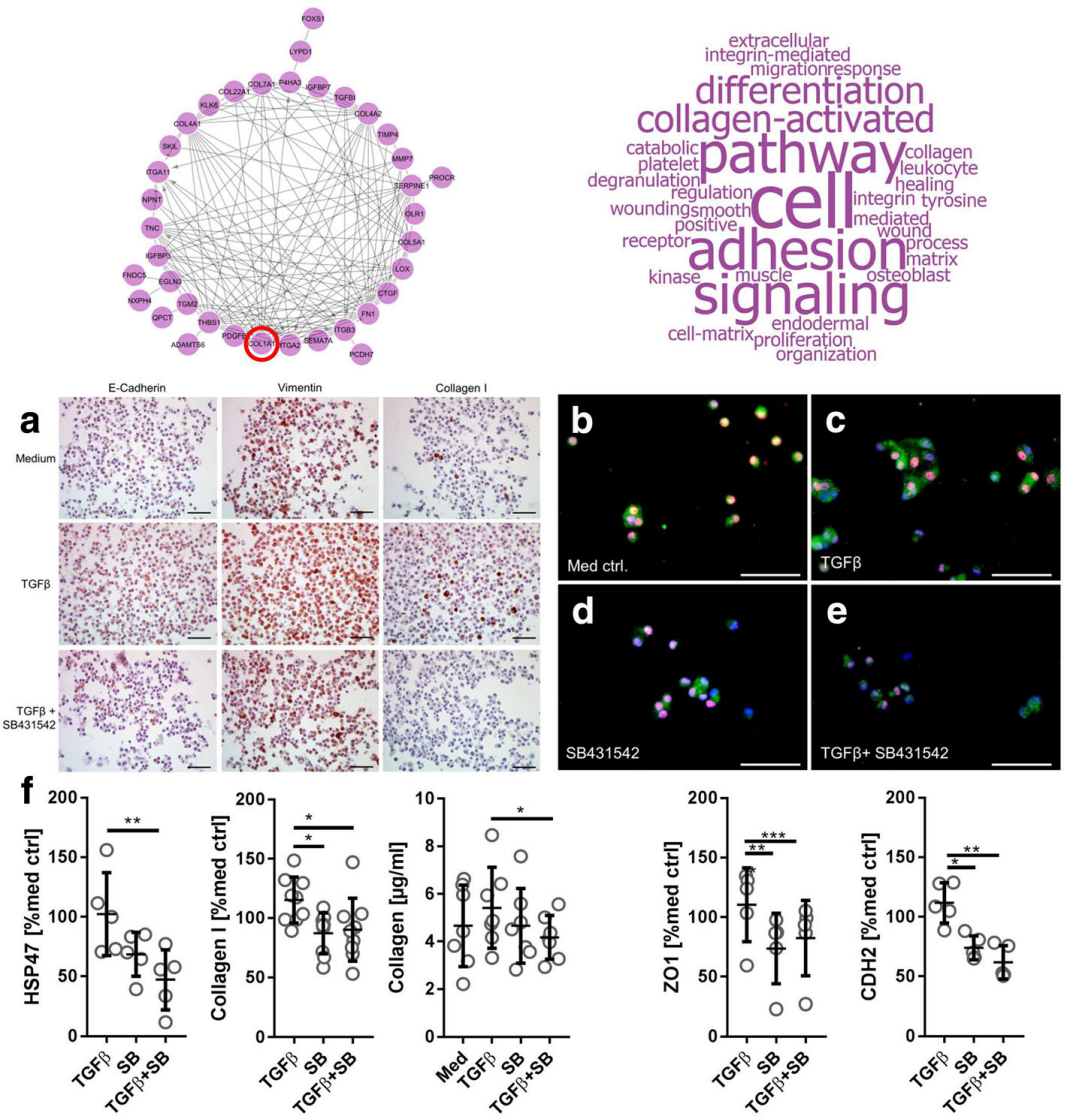
TGFβ regulates E-Cadherin, Vimentin and Collagen I on protein level in hAECII. Immunocytochemistry (**a**) was used to assess protein expression of E-Cadherin, Vimentin and Collagen I in paraffin-embedded hAECII from cell culture experiments (*N* = 2). Representative image shown with a scale bar = 100 μm. Red colour indicates positive signals. **b-e** Double immunofluorescence staining was used to show an up-regulated collagen metabolism in AECII lineage by TGFβ-stimulation. The thyroid transcription factor I (TTF-1) of the surfactant molecules was targeted as a proof of AECII lineage and the collagen chaperon HSP47 was used to display an elevated collagen synthesis. Nuclei are stained by DAPI (blue). TTF1 was visualized with a TRITC-conjugated secondary antibody (red) and HSP47 with an Alexa488-conjugated (green) secondary antibody. Exemplary image of 3 biological replicates with scale bar =50 μm show the expression of HSP47 in medium control **b**, with 5 ng/ml TGFB1 **C**, with 10 μm SB431542 (**d**) or both, TGFB1 and SB431542 (**e**). For means of cross-validation of results from ICC and IF, In-Cell Western analysis was conducted (**f**)s to investigate regulation of Collagen I, HSP47, N-Cadherin and ZO1 on protein level in hAECII . Cells were seeded in 96 well plates and stimulated for 48 h. Protein expression of Collagen I, HSP47, ZO1 and N-Cadherin/CDH2 was detected by means of ICW assay with primary antibodies against the targets and near-infrared conjugated secondary antibodies. To-Pro3 was used to stain all cells as loading control. Data is shown as semi-quantitative fluorescent signal normalized to total amount of cells, corrected for background signal and normalized to medium controls. N = 5 (HSP47, ZO1, N-Cadherin) or *N* = 9 (Collagen I). Semi-quantitative measurement of collagen produced by hAECII as stained by SiriusRed (*N* = 7)

Beside a migratory phenotype, as indicated by actin cytoskeleton remodeling, EMT is characterized by increased expression of mesenchymal markers and reduced expression of epithelial markers. We therefore strived to investigate the change of different EMT markers in hAECII upon TGFβ stimulation (Fig. 4). Cultivated hAECII expressed on a baseline level E-Cadherin, but also Vimentin and at a low level, Collagen I after 48 h of incubation (Fig. 4a). Addition of TGFβ resulted in a dramatic increase of Vimentin expression as well as intracellular Collagen I. Surprisingly, the expression of E-Cadherin did not seem to be strongly affected by TGFβ1 stimulation. Co-stimulation of hAECII with TGFβ1 and SB431542 reduced the Vimentin signal and almost completely abolished expression of Collagen I but left E-Cadherin expression unaltered. To finally prove the hAECII lineage and that hAECII are truly capable of synthesizing Collagen I, double immuno-fluorescence stainings were conducted (Fig. 4b-e). The AECII lineage was targeted by TTF-1, a transcription factor that specifically binds to promoter regions and regulates the surfactant molecules SPA, SPB, SPC and Clara Cell Secretory Protein (CCSP), which are only found to be expressed in AECII [19, 24]. An elevated collagen synthesis circuit was investigated by targeting the Heat Sock Protein 47 (HSP47), a collagen chaperon that is crucial for correct folding of collagen molecules [20] as well as is supposed to play a role in fibrosis [25]. TTF-1 positive hAECII cells expressed HSP47 (Fig. 4b) at a baseline level under culture conditions. Once TGFβ was added to the cells, an increase of HSP47 expression in these cells was observed (Fig. 4c). Furthermore, inhibition of the canonical TGFβ signaling cascade by SB431542 resulted in a distinct reduction of HSP47 (Fig. 4d-e). These findings were further validated by In-Cell Western Blot experiments which targeted native Collagen I, HSP47, ZO-1 and N-Cadherin/CDH2 via antibodies on or in hAECII seeded in 96 well plates (Fig. 4f). Both, Collagen I and HSP47 were found on an elevated level above the medium control baseline and the TGFβ-induced up-regulation was efficiently blocked by inhibiting the pathway. To our surprise, Zonula Occludens 1 (ZO-1), which is an integral member of the tight junctions, was rather found to be induced by TGFβ stimulation. N-Cadherin/CDH2 as a marker for mesenchymal differentiation did show an elevated expression upon TGFβ stimulation which was significantly abrogated by inhibition of the pathway. Cross-validation of the hAECIIs capability to produce Collagen was done by quantification using SiriusRed and did result in increased intracellular collagen upon TGFβ stimulation which was again significantly reduced by pathway inhibition (Fig. 4f).

### A consensus list of genes from the TGFβ fingerprint of AECII is differentially expressed in IPF patient lungs

**Fig. 5:**
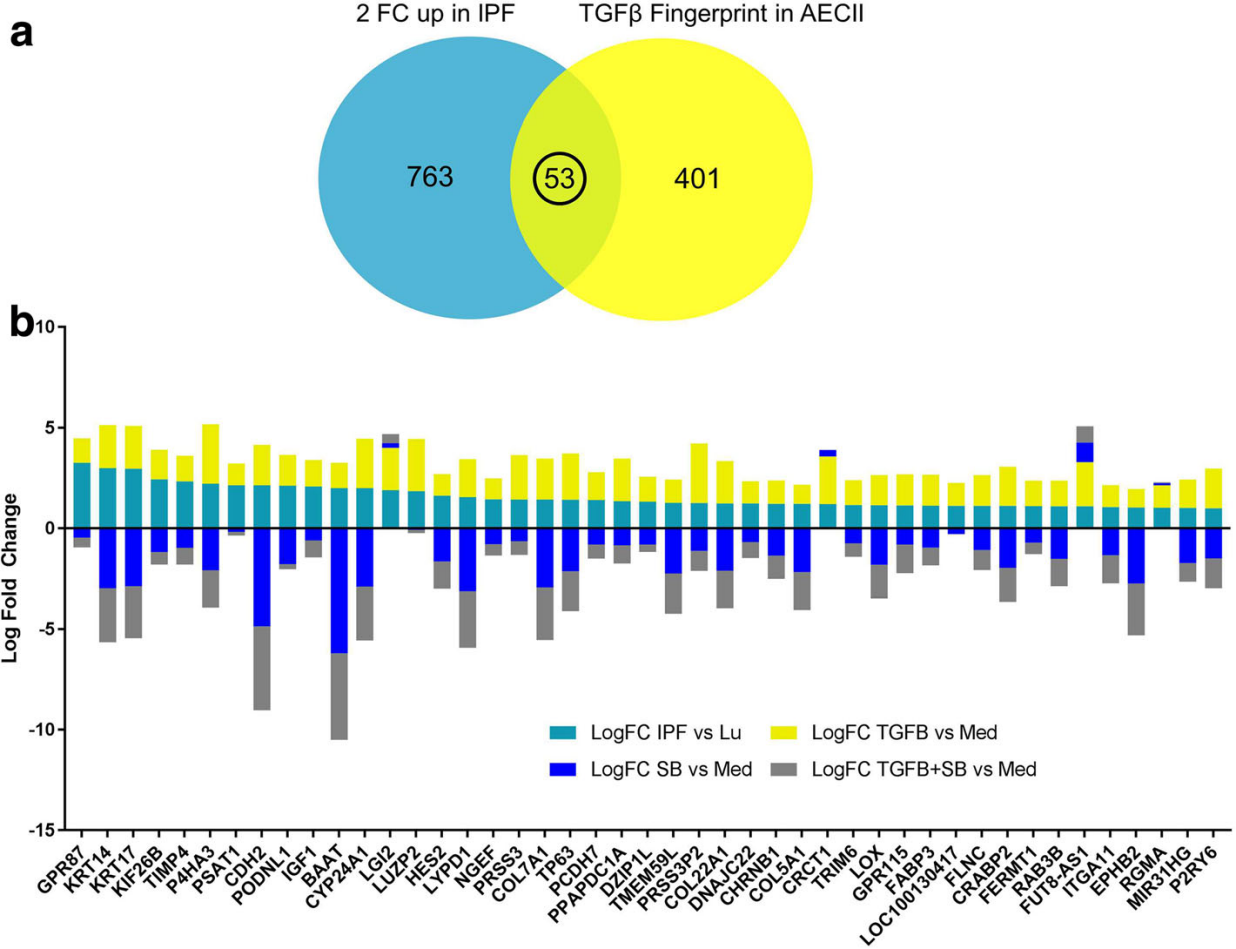
Consensus between the TGFβ *fingerprint* and up-regulated genes in tissues of IPF patients. An external dataset (GSE10667) of microarray results from the tissues of 23 IPF patients and 15 healthy controls was used to investigate the potential overlap with genes from the *TGFβ fingerprint* in hAECII. 53 genes were shared between both datasets (**a**). The Log Fold Changes of these genes (IPF compared to normal lung or to the unstimulated hAECII control, respectively) are depicted as bar charts with the respective direction (**b**)

To further investigate the clinical relevance of previous results, the *TGFβ fingerprint* expression profile was compared to gene expression data from IPF patient lungs. Here, the significantly up-regulated genes from IPF tissues compared to normal lung tissues (23 IPF vs 15 lungs) were analyzed by Venn diagram with the *TGFβ fingerprint* (Fig. 5a). A consensus list of genes from both datasets was derived and the Log Fold Change values for each gene and experimental condition are shown in Fig. 5b. An overlap of 53 genes was found to be up-regulated in IPF as well as by TGFβ in hAECII. All of these genes displayed a regulation in the same direction and the majority of these genes were successfully reversed in their expression upon pathway inhibition. Even more, the consensus genes exhibited a significant enrichment from the GSEA Hallmark gene sets (Epithelial-Mesenchymal-Transition, *q* = 0.00155 and Myogenesis, *q* = 0.019) as well as Reactome pathways (Collagen Formation, q = 0.013 and Extracellular Matrix Organization, *q* = 0.022).

## Discussion

The origin of the ECM secreting cell(s) in the pathogenesis of IPF has been a focus of many studies and a general consensus on the involvement of the TGFβ pathway or other developmental pathways have been acknowledged [26]. AECII have been hypothesized to contribute to IPF pathology by EMT [10] and acquire accordingly the potential to synthesize and secrete ECM molecules, thereby directly influencing the local fibrotic process. Studies using either bronchial-epithelial cells [27] or A549 lung cancer cells [15] to investigate the effect of TGFβ, suggested that EMT in these cells is SMAD-dependent and further studies employing AECII from mice could show that these cells undergo EMT in response to bleomycin in vivo [28] or in vitro [29], assuming a relevance during IPF pathogenesis. Immunohistochemistry studies on human IPF tissues localized vimentin-expressing epithelial cells in the area of fibroblast foci [30] and furthermore the presence of the EMT molecules smooth muscle actin/ACTA2 or TWIST [31], thereby suggesting EMT taking place, but leaving the origin of the collagen-secreting cell elusive. These findings from human samples could not be reproduced using lineage-tracing experiments in mice [32]. Hence, the present study used primary hAECII to test this hypothesis.

By investigating the transcriptomic response to TGFβ, we show that hAECII significantly up-regulate expression modules that suggest (trans)-differentiation processes taking place in hAECII and even more distinct processes as wound healing and cytoskeleton involvement. A comparable GO-enrichment pattern has been observed in IPF tissues from exacerbating patients [33] or, with regards to differentiation and developmental GO-terms, as differentially enriched in IPF compared to hypersensitive pneumonia [34]. As TGFβ-stimulated hAECII show transcriptional signatures resembling those observed in IPF samples and suggest a transdifferentiation process, these changes were furthermore accompanied by a pronounced actin-cytoskeleton remodeling and morphological change which is regarded as a hallmark feature of EMT [12, 35]. Interestingly, not all classical features of EMT were observed in stimulated hAECII. Although consistent induction of actin-cytoskeleton remodeling was observed, as well as respective gene sets and induction of mesenchymal markers on protein level, E-Cadherin or tight-junction proteins were not repressed as expected. However, different forms of EMT have been described and a transient form of EMT which retains E-Cadherin expression and expresses alpha smooth muscle actin as well as N-Cadherin and Collagen I has been designated Type 2 EMT and advocated to be mainly involved in fibrosis and wound healing processes [36]. This is in line with our observation and suggests a Type 2 EMT taking place in a TGFβ-dependent manner in primary hAECII. Furthermore, these cells acquire the capability to produce collagen I, which is known to be aberrantly expressed in lungs of IPF patients [37]. Also the collagen chaperon HSP47, which has previously been shown to play a role during IPF exacerbations [38], was found to be induced by TGFβ in hAECII, thereby mimicking the processes taking place in patient lungs during IPF.

To our knowledge, this is the first study to directly link a TGFβ-dependent Type 2 EMT in hAECII, even so only transiently, with a concurrent gain of collagen production and thereby propose to add this additional cell type to the discussed pool of myofibroblast-resembling cells that are sources of ECM deposition in tissue fibrosis. Data from animal model suggest up to 30% of cells from epithelial origin contribute to the S100A4 positive “fibroblast” population during IPF [14]. A recent study investigated the effect of KLF4 on EMT during fibrosis in mice and human alveolar epithelial cells and came to similar results about EMT, but failed to describe the analyzed primary cell population properly as these were purchased from a vendor [39]. Furthermore a recent study investigating the effect of ResolvinD_1_ during TGFβ-induced EMT in AECII found that ResolvinD_1_ itself promotes trans-differentiation of these cells and seem to interfere with a TGFβ-mediated effect in AECII. These results are perfectly in line with our in vitro observations and strengthen the observation that primary human AECII are capable of trans-differentiation [40]. The cross-validation of the TGFβ-fingerpint of hAECII with significantly up-regulated genes from an independent dataset of patient tissues pointed out the potential relevance for the clinics: The consensus set of genes revealed a similarity in the direction of regulation as well as the fact, that these genes are successfully reversed in their regulation upon pathway inhibition. This could give constructive insights into treatment options for IPF patients as inhibiting the TGFβ-pathway successfully reversed or impeded the Type 2 EMT of hAECII. The significance of TGFβ-pathway inhibition was already translated into clinical use by application of Pirfenidone [41] or in animal models of IPF [42]. However, the outlined data would propose a possible benefit applying specific TGFβ-pathway inhibitors since the way of pathway inhibition by Pirfenidone is not completely understood yet [43]. It has not escaped our attention that cultured hAECII seemed to behave in vitro already in a pro-EMT state with a baseline collagen expression per se, which might be influenced by culturing on uncoated cell culture surfaces. Thus we assume that the observed phenomena could be even more distinct if hAECII were cultured on coated surfaces that resemble more their original niche in the alveolus. Furthermore we cannot exclude that the observed effects are true in a chronic model of IPF or over longer periods of experimental time. It is conceivable that the primary cells might acquire the Type 2 EMT state only transiently and reverse their phenotype again. In addition, in vitro experimental work in the field of IPF shows a variety of applied TGFβ concentrations. Early studies used as much as up to 5 ng/ml [15, 16, 29] while other studies applied lower ranges (1–2 ng/ml). It is very likely that the experimental time-frame as well as the applied concentrations might influence the pronunciation of each applied read-out technique as well as target molecules. Some observations that are true for a higher concentration of TGFβ might result in a less distinct level of regulation by lower concentrations or prolonged incubation times.

## Conclusions

hAECII undergo in vitro a phenotypic change upon TGFβ-stimulation and acquire the capability to produce collagen as well as display phenotypic changes on gene expression and protein level that resemble Type 2 EMT. Hence, these cells might contribute to the pool of collagen-secreting cells in the alveolar compartment during wound healing processes or fibrotic reactions.

## Additional files

Additional file 1: **Table S4.** Patient data. Spreadsheet containing patient characteristics (gender, age at diagnosis, COPD status, packyears and smoking-status). (XLSX 10 kb)

Additional file 2: **Figure S1.** Immunocytochemistry was used to assess protein expression of TTF1, pro-SPC and Podoplanin in paraffin-embedded hAECII directly after magnetic bead separation. Representative images shown with a scale bar = 100 μm are shown. The median TTF1-positivity from 11 different extractions was 90% (A), 95% median positivity for pro-SPC (B) and 2.5% median positivity for Podoplanin (C).. Red colour indicates positive signals. (JPG 1152 kb)

Additional file 3: **Table S1.** Results from 1 W-ANOVA for regulation between experimental groups. Spreadsheet containing microarray data from statistical analysis. (XLSX 2864 kb)

Additional file 4: **Table S2.** Results from Reactome FIViz module analysis. Spreadsheet containing results from network analysis using Cytoscape and the Reactome FIViz app for discovery of interaction modules. (XLSX 10 kb)

Additional file 5: **Table S3.** Enriched GO terms. Spreadsheet containing results from network analysis using Cytoscape and the Reactome FIViz app for discovery of interaction modules. Significant enriched GO terms for Biological Process for each module are presented. (XLSX 79 kb)

## Abbreviations

AECII: Alveolar epithelial cells type II
DPLD: Diffuse parenchymal lung diseases
ECM: Extracellular matrix
EMT: Epithelial-Mesenchymal-Transition
hAECII: Human alveolar epithelial cells type II
IPF: Idiopathic Pulmonary Fibrosis
TGFβ: Transforming Growth Factor β
