# Supplementary figures and images for: Human alveolar epithelial cells type II are capable of TGFβ-dependent epithelial-mesenchymal-transition and collagen-synthesis
Source: Respir Res. 2018 Jul 24;19:138. doi: 10.1186/s12931-018-0841-9 (PMC6056940; doi:10.1186/s12931-018-0841-9)

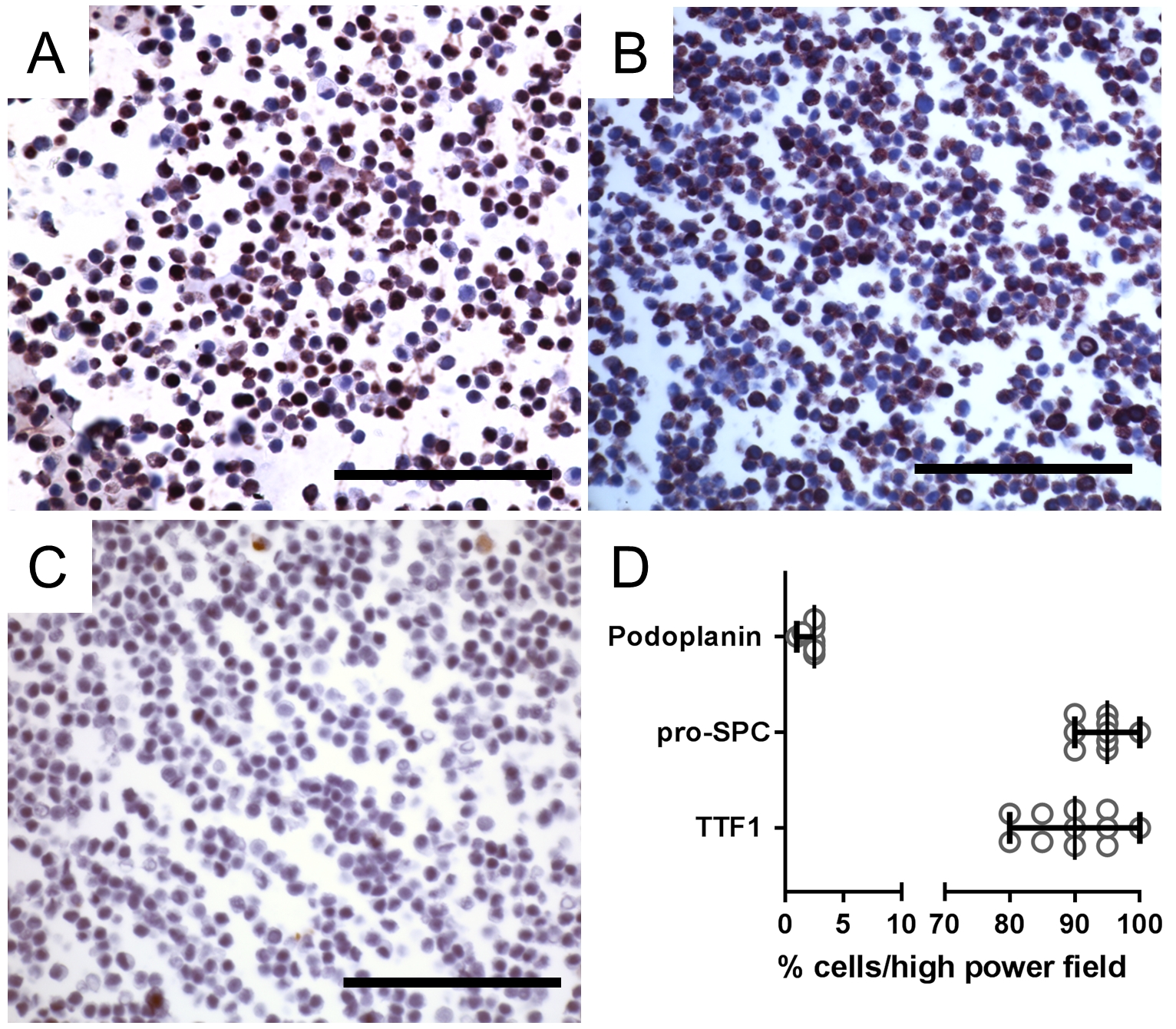

Supplement: Supplementary file 2 — Figure S1. Immunocytochemistry was used to assess protein expression of TTF1, pro-SPC and Podoplanin in paraffin-embedded hAECII directly after magnetic bead separation. Representative images shown with a scale bar = 100 μm are shown. The median TTF1-positivity from 11 different extractions was 90% (A), 95% median positivity for pro-SPC (B) and 2.5% median positivity for Podoplanin (C).. Red colour indicates positive signals. (JPG 1152 kb) [file 12931_2018_841_MOESM2_ESM.jpg]
